# Supplementary material for: Association of Health Insurance Status with Outcomes of Sepsis in Adult Patients: A Retrospective Cohort Study
Source: Int J Environ Res Public Health. 2021 May 27;18(11):5777. doi: 10.3390/ijerph18115777 (PMC8198413; doi:10.3390/ijerph18115777)
Supplement: Supplementary file 1 [file ijerph-18-05777-s001.zip › ijerph-1220363-supplementary.pdf]

Table S1. Survival outcomes by participating hospitals in the full study cohort.

**Hospital 1**				
Variables	Total	National Health insurance	Medical Aid	*P*-value
n (%)	774 (100.0)	739 (95.5)	35 (4.5)	
In-hospital mortality	176 (22.7)	167 (22.6)	9 (25.7)	0.68
28-day mortality	151 (19.5)	141 (19.1)	10 (28.6)	0.19
90-day mortality	196 (25.3)	186 (25.2)	10 (28.6)	0.69

Hospital 2				
Variables	Total	National Health insurance	Medical Aid	*P*-value
n (%)	1,194 (100.0)	1,132 (94.8)	62 (5.2)	
In-hospital mortality	333 (27.9)	321 (28.4)	12 (19.4)	0.15
28-day mortality	232 (19.4)	224 (19.8)	8 (12.9)	0.19
90-day mortality	367 (30.9)	355 (31.5)	12 (19.4)	0.05

Hospital 3				
Variables	Total	National Health Insurance	Medical Aid	*P*-value
n (%)	558 (100.0)	458 (82.1)	100 (17.9)	
In-hospital mortality	131 (23.5)	110 (24.0)	21 (21.0)	0.60
28-day mortality	104 (20.8)	91 (22.2)	13 (14.6)	0.12
90-day mortality	134 (32.5)	114 (32.9)	20 (30.3)	0.78

Table S2. Hospital costs during hospitalization in a participating hospital by patient's health insurance status.

**Variables**		**Total**	**National Health Insurance**	**Medical Aid**	***P*-value**
Total cost	Dollar ($)	4,670 (2,598-8,542)	4,683 (2,664-8,586)	4,539 (2,248-8,427)	0.628
	Won (₩)	5,237,581 (2,913,925-9,580,320)	5,252,155 (2,987,377-9,629,096)	5,090,698 (2,520,749-9,450,589)	
Actual cost	Dollar ($)	902 (389-1,711)	1,046 (543-1,945)	184 (65-536)	< 0.001
	Won (₩)	1,011,595 (436,038-1,919,300)	1,172,650 (608,688-2,181,795)	206,600 (73,164-601,223)	

Data were presented as median (interquartile range). Actual cost means the amount of medical cost actually paid by the patient, applying insurance coverage and copayment by insurance type.
